# Assessment of immune response to repeat stimulation with BCG vaccine using in vitro PBMC model

**DOI:** 10.1186/1476-8518-8-3

**Published:** 2010-05-28

**Authors:** Rajpal S Kashyap, Aliabbas A Husain, Shweta H Morey, Milind S Panchbhai, Poonam S Deshpande, Hemant J Purohit, Girdhar M Taori, Hatim F Daginawala

**Affiliations:** 1 Biochemistry Research Laboratory, Central India Institute of Medical Sciences, 88/2 Bajaj Nagar, Nagpur-440010, India; 2 Environmental Genomic Unit, National Environmental Engineering Research Institute. Nehru Marg, Nagpur-440020, India

## Abstract

**Background:**

Tuberculosis (TB) is one of the most prevalent cause of death due to a single pathogen. Bacillus Calmette Guérin (BCG) is the only vaccine available for clinical use that protects against miliary TB; however, this vaccine has shown variable levels of efficacy against pulmonary TB. In India, a single dose of BCG vaccine is given and there are few countries where repeated doses of BCG are given. The incidence of TB in India is very high inspite of primary vaccination in neonatal period and therefore requires consideration for repeated immunization.

**Methods:**

To improve BCG immunogenicity, we have evaluated specific antimycobacterial immune responses (anti-BCG IgG and IFN-γ), T cell activity-ADA, CD4 and CD8 T cell count, and CD4/CD8 ratio in a peripheral blood mononuclear cells (PBMC) model using boost immunization protocols with the BCG vaccine. PBMC were induced with a **repeat **dose of BCG at 24 and 72 hrs of cell culture.

**Results:**

At the end of the experimental time, supernatant was collected to estimate anti-BCG IgG titer, interferon γ, ADA activity, CD 4 and CD8 T cell count, and CD4/CD8 ratio. We demonstrated that PBMC induced with a repeat dose of BCG showed an increased specific anti-mycobacterial immune responses, T cell activity, and ADA activity as compared to PBMC induced with BCG alone or without BCG induction.

**Conclusion:**

The repeat BCG stimulation of PBMC obtained from BCG vaccinated individuals shows enhanced immune activation with respect to increased anti-BCG titre, IFN-γ and ADA activity without concomitant increase in CD4 and CD8 cells. This study provides some basic data in future experiments in animal models with respect to repeat BCG vaccination.

## Introduction

In India, tuberculosis (TB) is the greatest cause of death due to a single pathogen. Bacillus Calmette-Guerin (BCG) is the only vaccine available for clinical use and is one of the most widely used vaccines, being both inexpensive and safe [1,2]. Despite early success, the BCG vaccine has had a limited effect against the incidence of TB in the developing world. Various clinical trials have demonstrated that BCG showed variable levels of efficacy against pulmonary TB. For example, a major trial in the United Kingdom showed >75% protection [3]; however, trials in south India and Malawi demonstrated that BCG failed to protect consistently against pulmonary TB [4,5]. The reasons for this have been a matter of debate and this indicates an urgent need for more effective vaccines to decrease the incidence of tuberculosis.

Current research interest has been directed towards improving the immunogenicity of BCG [6]. Recently, recombinant BCG expressing ESAT-6 was found to confer enhanced protection against tuberculosis compared to normal BCG [7]. Similarly, it has been demonstrated that the recombinant BCG vaccine expressing the *Mycobacterium tuberculosis *30-kDa major secretory protein induced greater protective immunity against tuberculosis than the conventional BCG vaccine [8]. A different approach is the heterologous expression of cytokines in BCG [9,10] or of bacterial proteins, such as *Listeria monocytogenes *listeriolysin, which improves the capacity to stimulate antimycobacterial T-cell responses [11,12]. An interesting complementary approach to improving BCG immunogenicity is its combination in prime-boost immunization protocols [3,5]: priming with a DNA vaccine that expresses antigen 85B and boosting with BCG improves the protective efficacy in a murine *M. tuberculosis *challenge model [8]. In spite of all these efforts, no new TB vaccine has been developed in the last 85 years as an alternative to BCG. This suggests that instead of developing several new molecules, a focus can be placed on the improvement of the current BCG vaccination protocol, which is the objective of our studies.

There are some countries that give repeated doses of BCG vaccine. For example, Turkey gives BCG immunization four times: during infancy at two months after birth, at six to seven years of age (first grade), at eleven to twelve years of age (fifth grade), and sixteen to seventeen years of age (high school) [13]. In India, a single dose of BCG vaccine is given within one week of the birth of child. The incidence of TB in India is very high and repeated immunization is needed as it is done in other countries. However, it is very difficult to start giving repeated doses of BCG in India without any experimental studies. The aim of the present study is to investigate the immune responses to repeated stimulation of PBMC's from BCG vaccinated healthy volunteers with BCG vaccine.

## Materials and methods

### Study Subjects

Participants were recruited for this study under protocols approved by the ethics committee of Central India Institute of Medical sciences (CIIMS), Nagpur, India and enrolled after obtaining informed consents. All the subjects aged 18-45 years were recruited having no history of pulmonary illness, tuberculosis, seronegative for HIV and HBV and had been vaccinated with BCG.

### PBMC model

**Figure 1:**
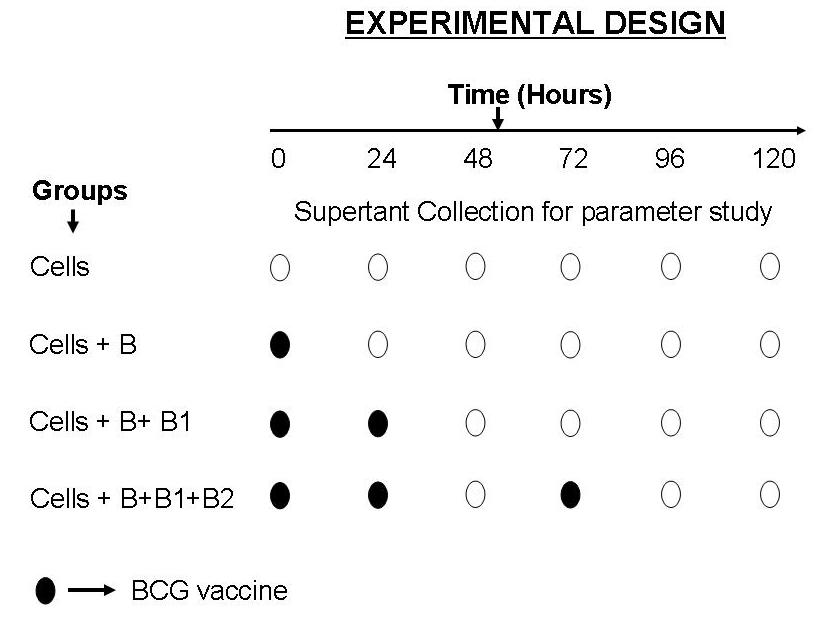
**Schematic representation of experimental design**.

PBMCs were separated from whole blood of healthy volunteers (n = 15) included in this study by density gradient centrifugation using the Ficoll Histopaque method. BCG vaccine (Moscow Strain) was obtained from Serum Institute of India, Pune and stored at 4-8°C. Prior to use, vaccine was reconstituted in sterile saline. After counting, the cells were cultured in RPMI-1640 medium keeping the concentration at 2 × 10^5 ^cells/well and were induced with the BCG vaccine (10 μl/ml or 10^4 ^CFU/ml). The cells not induced with BCG vaccine were taken as controls. Induced cells were then incubated for 0, 4, 24, 48, 72, 96, and 120 hrs in a CO_2 _incubator. Booster doses of BCG (10 μl) were given after 24 and 72 hrs. The cells were then taken out from the incubator and were centrifuged for 10 mins at 1000 rpm. Supernatant was separated and was analyzed for anti-BCG IgG titer, adenosine deaminase activity, and interferon γ levels. The pellet was suspended in phosphate buffer saline (pH 7.2) and was used for flow cytometry analysis to determine CD 4 and CD 8 T cell count. The detailed experimental sketch is given in figure 1.

### Anti-BCG (IgG) estimation

An in-house developed Indirect ELISA method was employed using a BCG vaccine. (Bacillus Calmette Guerin strain, Serum Institute Of India Ltd, Pune, India) to estimate the Anti-BCG (IgG) titer/level. Briefly the 96-well microtiter plate (MaxisorpImmunoplate, Nalge Nunc International, Naperville, III.) were coated with 10 ng of BCG (diluted in sterile saline). After 3 hours of incubation at 37°C, the plates were washed and blocked with 0.25% BSA in Phosphate buffered saline (PBS) pH 7.40. After 60 minutes of incubation, plates were washed once and kept overnight at 4°C. Next day the plates were incubated with supernatant (1:400 diluted) in PBS. After 45 minutes of incubation plates were washed and incubated with rabbit anti-mouse IgG, HRP conjugate (1:10,000) for 45 minutes. For color development substrate Tetramethyl benzidine in hydrogen peroxide (TMB/H2O2) was added and incubated for 10 min. The reaction was stopped by adding 2.5N sulphuric acid and the optical density of plates was read at 450 nm.

### Interferon γ (gamma)

IFN-γ was measured by an enzyme linked immunosorbant assay (ELISA) according to the manufacturer's instructions (Bender Med System, Austria). In brief, anti IFN-γ monoclonal coating antibody was adsorbed onto the microwells. After two hours of incubation at room temperature, the wells were washed and blocked with 0.5% BSA in phosphate buffer. After one hour of incubation at room temperature, supernatant followed by biotin-conjugated anti-cytokine antibody was added to the coated wells. After another two hours of incubation, streptavidin-HRP (horseradish peroxidase) was added to the wells. After one hour of incubation, streptavidin-HRP was removed by washing and substrate solution reactive with HRP was added to the wells. A colored product was formed in proportion to the amount of cytokine present in the sample. The reaction was terminated by the addition of 4 N sulphuric acid and the absorbance was measured at 450 nm.

### Adenosine deaminase (ADA)

ADA activity in the supernatant was determined at 37°C according to the method of Guisti and Galanti [14] based on the Berthlot reaction, in which there is the formation of colored indophenol complex from ammonia liberated from adenosine and quantified spectrophotometrically (U.V. Visible spectrophotometer, Systronic-Model). One unit of ADA is defined as the amount of enzyme required to release 1 m mol of ammonia/min from adenosine in standard assay conditions. Results were expressed as units per litre per minute (U/L/min). The assays were performed in triplicate and blind to the diagnosis.

### Flow cytometric analysis

After performing experiments, the PBMCS obtained were suspended in PBS and subjected to flow cytometry analysis to determine CD 4 and CD 8 T cell level/cell count. Flow cytometry was performed on a fluorescence activated cell sorter (FACS-SCAN) instrument (Becton Dickinson Biosciences) Briefly the cells were centrifuged and 50 ul of sediment was stained using 5 ul antibodies attached to specific flourochromes against CD8-PER CP, CD4-PE and CD-3 -FITC (all from Becton Dickinson Biosciences) and incubated for 30 minutes at room temperature. The cells were again washed and resuspended in 1 ml saline and subjected to flowcytometry. Cells collected using flow cytometry on a FACS were analyzed using FlowJo software by gating on the lymphocyte population in forward scatter (FSC) and side scatter (SSC). The gate was set around the lymphocytes to exclude other cells from analysis. Routinely 10,000 cells per tube were counted.

## Results

**Figure 2:**
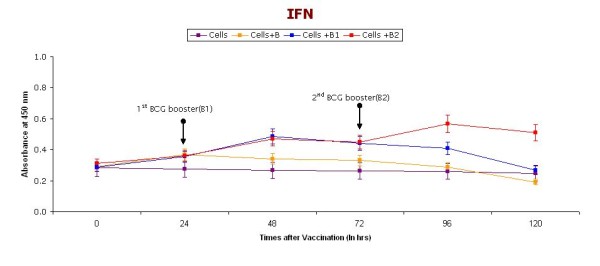
**IFN-γ levels in supernatants (collected at different time intervals) of short-term cultures of PBMC induced with BCG vaccine**. Booster doses of BCG (2.5 μl) were given (indicated with arrow) after 24 & 48 hrs. Cells represent control group without vaccination, Cells+ B represent group receiving BCG vaccination without any booster dose, Cells + B1 represent group receiving only one booster dose of vaccine, Cells + B2 represents groups receiving two Booster doses of BCG vaccination.

Figure 2 shows the IFN-γ levels in supernatants collected at different time points (0, 24, 48, 72, 96, and 120 hrs) of short-term cultures of PBMC induced with BCG vaccine. Booster doses of BCG (2.5 μl) were given after 24 and 48 hrs. An initial increase in IFN-γ secretion in response to BCG (single dose) was noted until 24 hrs and a decrease started after 24 hrs. However, after giving a repeat dose of BCG at 24 and 72 hrs, the IFN-γ levels increased and were much higher compared to the preboost and without BCG groups of cultures.

**Figure 3:**
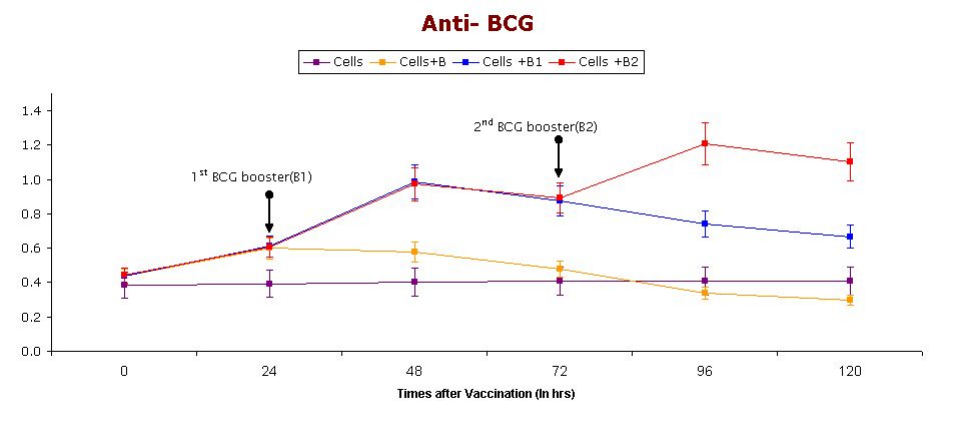
**Anti BCG (IgG) levels in supernatants (collected at different time intervals) of short-term cultures of PBMC induced with BCG vaccine**. Booster doses of BCG (2.5 μl) were given (indicated with arrow) after 24 & 48 hrs. Cells represent control group without vaccination, Cells+ B represent group receiving BCG vaccination without any booster dose, Cells + B1 represent group receiving only one booster dose of vaccine, Cells + B2 represents groups receiving two Booster doses of BCG vaccination

Cellular immune responses are key to an effective protection against TB. Figure 3 shows the BCG specific antibody mediated response (anti-BCG titer) in supernatants collected at prespecified time intervals from different experimental groups along with controls. Our data indicate that PBMC induced with booster doses of BCG show increased specific anti-mycobacterial immune responses (anti-BCG IgG) compared to preboost group and control group.

**Figure 4:**
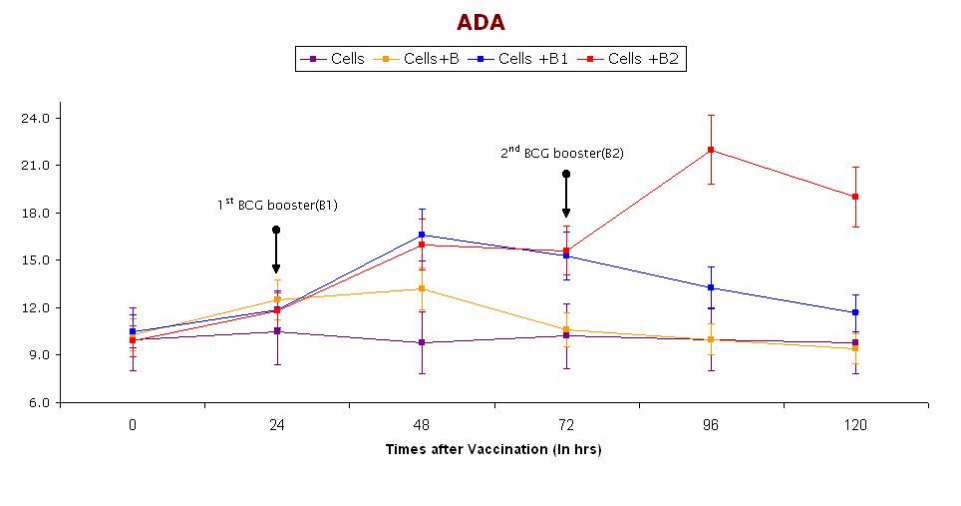
**Level of ADA in supernatants (collected at different time intervals) of short-term cultures of PBMC induced with BCG vaccine**. Booster doses of BCG (2.5 μl) were given (indicated with arrow) after 24 & 48 hrs. Cells represent control group without vaccination, Cells+ B represent group receiving BCG vaccination without any booster dose, Cells + B1 represent group receiving only one booster dose of vaccine, Cells + B2 represents groups receiving two Booster doses of BCG vaccination

ADA activity in supernatants was determined by the Guisti and Galanti method. Figure 4 shows the ADA levels in supernatants collected at different point (0, 24, 48, 72, 96, and 120 hrs) of the short-term cultures of PBMC induced with BCG vaccine. ADA activity was found to increase immediately after giving the first dose, but decreased again after 72 hrs. However, the activity increased at the time when booster dose was given, but decreased again after 96 hrs.

**Figure 5:**
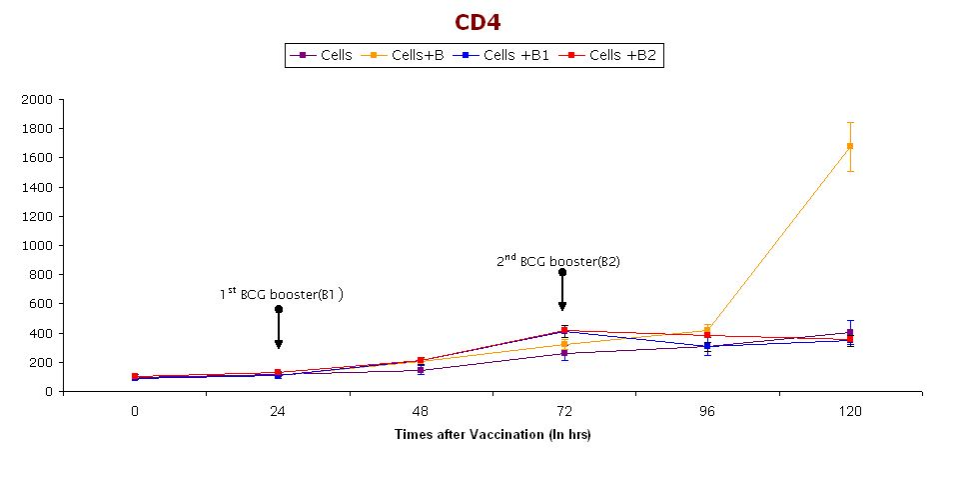
**CD4 cells (at different time intervals) in short-term cultures of PBMC induced with BCG vaccine**. Booster doses of BCG (2.5 μl) are given (indicated with arrow) after 24 & 48 hrs. Cells represent control group without vaccination, Cells+ B represent group receiving BCG vaccination without any booster dose, Cells + B1 represent group receiving only one booster dose of vaccine, Cells + B2 represents groups receiving two Booster doses of BCG vaccination

**Figure 6:**
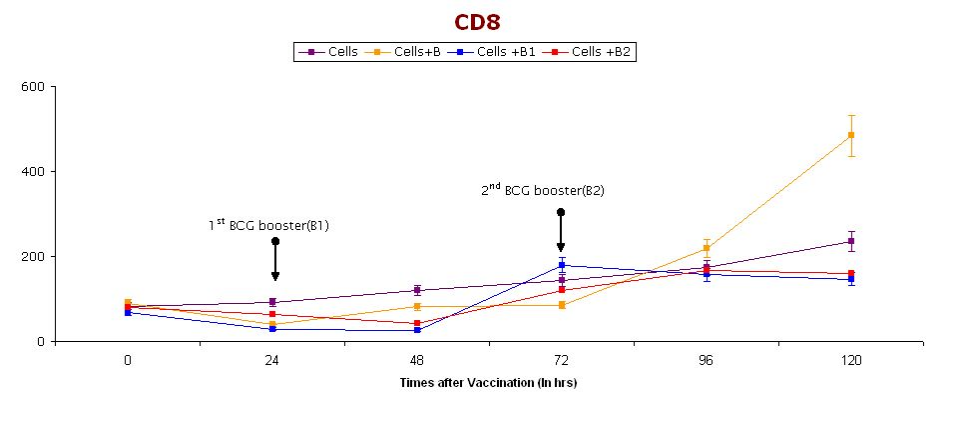
**CD8 cells (at different time intervals) in short-term cultures of PBMC induced with BCG vaccine**. Booster doses of BCG (2.5 μl) are given (indicated with arrow) after 24 & 48 hrs. Cells represent control group without vaccination, Cells+ B represent group receiving BCG vaccination without any booster dose, Cells + B1 represent group receiving only one booster dose of vaccine, Cells + B2 represents groups receiving two Booster doses of BCG vaccination.

Figure 5 and Figure 6 shows CD 4 and CD8 levels in samples collected at pre-specified time intervals from the preboost, after booster, and without booster groups. However, no significant change was observed in both CD4 and CD8 levels after booster dose.

## Discussion

Various molecules have been previously investigated, and currently, many molecules are in process for the development of improved vaccine for tuberculosis [14,15]. Despite these efforts, not a single vaccine has been developed in the last 85 years. The BCG vaccination is still used in almost every part of the world for protection against TB, however the outcome is variable. There are few studies that indicate that BCG gives short term protection [16]. Like some other well known vaccines which give better protection after a repeat dose, it was postulated by us that repeat dose of BCG vaccine may also enhance immunogenicity. Therefore, to study this, we have evaluated an anti-BCG IgG titer, interferon γ response, and ADA activity in a peripheral blood mononuclear cells (PBMC) model with repeat dose of BCG vaccine.

Our data show that induction of repeat dose of BCG in the PBMC model increased specific antimycobacterial immune responses (anti-BCG IgG and IFN-γ, T cell activity-ADA). In other words we have shown that culturing of human PBMC's with repeat dose of BCG shows increased memory response to previous immunization. Recently P M Udani has raised a question of whether repeat doses are needed in countries where the burden of TB is high [16]. Our initial work suggests that the efficacy of BCG may improve with repeat doses. Secondly, these results may be helpful in designing future experiments in animal models with respect to a booster approach [17]. Ultimately, with the help of all experimental evidences, India may start repeat immunizations of BCG and may reduce the burden of TB in future generations.

Three major questions arise while discussing the revaccination concept of the BCG vaccine. The first question is: what is the impact of repeated BCG vaccination on tuberculin skin test (TST) responses. Uyan et al [13] evaluated the TST response after BCG immunization in children and observed that the mean induration diameter increased after repeated BCG vaccination and simultaneously increased scar counts. From this study, it is confirmed that repeat BCG immunizations alter the TST response and therefore affect the use of TST in the diagnosis of TB infection in this group of children. Another important question is whether a repeat dose of BCG causes tuberculosis or not. We have searched the literature to answer this question and we have not found any evidence which suggests that repeat BCG immunization causes TB. In fact, Menzies et al have reported that a repeat BCG immunization is rarely the cause of TB infection [18]. Thirdly whether repeat BCG dose will give enhanced protection in humans even though, observed in invitro PBMC studies can only be decided after proper controlled animal model and clinical studies.

It is important to critically evaluate the results presented in this study which has some major limitations that needs to be evaluated while considering BCG revaccination. The data shows an increase in IgG in BCG stimulated PBMC, which is an encouraging finding. However, it will be very important to measure IgG response to non-related immunogen (control Ag) to rule out any non-specific immune response. Secondly, the risk of development of TB cannot be studied with the developed in vitro model and therefore the study requires the development of an animal model. The other limitation in the present model is inability to observe the immune response in PBMC after a particular time (120 hrs in our study). However, this preliminary study with a PBMC model provides valuable information for the design of appropriate animal models for further studies.

## Conclusion

The repeat BCG stimulation of PBMC obtained from BCG vaccinated individuals shows enhanced immune activation with respect to increased anti-BCG titre, IFN-γ and ADA activity without concomitant increase in CD4 and CD8 cells. This study provides some basics data in future experiments in animal models with respect to repeat BCG vaccination.

## Competing interests

The authors declare that they have no competing interests.

## Authors' contributions

RSK carried out the study planning, data collection, statistical analysis, data interpretation, literature search and manuscript preparation. AAH participated in the literature search, preparation of the manuscript and data interpretation. SHM carried out the animal cell culture and flow cytometer experiments and data collection. MSP carried out the cytokines and other biochemical parameters. PSD carried out the study designing of animal tissue cell experiments. HJP participated in the preparation of the manuscript and data interpretation. GMT provided assistance in preparation of the manuscript, data interpretation and study designing. HFD supervised the study design, statistical analysis, data interpretation, manuscript preparation and literature search. All authors read and approved the final version of the manuscript.
